# Distributed Deformation Monitoring for a Single-Cell Box Girder Based on Distributed Long-Gage Fiber Bragg Grating Sensors

**DOI:** 10.3390/s18082597

**Published:** 2018-08-08

**Authors:** Sheng Shen, Shao-Fei Jiang

**Affiliations:** 1 Department of Civil Engineering, Fuzhou University, Fuzhou 350108, China; cejsf@fzu.edu.cn; 2 Hebei Province Key Laboratory of Evolution and Control of Mechanical Behavior in Traffic Engineering Structure, Shijiazhuang Tiedao University, Shijiazhuang 050043, China

**Keywords:** deformation monitoring, distributed monitoring, single-cell box girder, long-gage strain, long-gage fiber Bragg grating, strain distribution, shear lag effect, shear action

## Abstract

Distributed deformation based on fiber Bragg grating sensors or other kinds of strain sensors can be used to monitor bridges during operation. However, most research on distributed deformation monitoring has focused on solid rectangular beams rather than box girders—a kind of typical hollow beam widely employed in actual bridges. The deformation of a single-cell box girder contains bending deflection and also two additional deformations respectively caused by shear lag and shearing action. This paper revises the improved conjugated beam method (ICBM) based on the long-gage fiber Bragg grating (LFBG) sensors to satisfy the requirements for monitoring the two additional deformations in a single-cell box girder. This paper also proposes a suitable LFBG sensor placement in a box girder to overcome the influence of strain fluctuation on the flange caused by the shear lag effect. Results from numerical simulations show that the theoretical monitoring errors of the revised ICBM are typically 0.3–1.5%, and the maximum error is 2.4%. A loading experiment for a single-cell box gilder monitored by LFBG sensors shows that most of the practical monitoring errors are 6–8% and the maximum error is 11%.

## 1. Introduction

Monitored deformation is used to evaluate the overall health and safety of in-service bridges and also to prevent abnormal states developing. Geodetic surveying using a digital level or total station has been widely applied to directly measure bridge deformation [1,2] because of its low cost and ease of operation. The main disadvantages of geodetic surveying are the possible obstruction to public traffic when the survey is ongoing and measurement error from manual observation. Recently, some automatic monitoring techniques, such as the global positioning system [3,4], displacement sensors [5], hydrostatic leveling system [6], and laser measurements [7], have been applied to measure bridge deformation. However, these sensors and systems may be disrupted by environmental factors including bad weather, accidental vibration, or satellite ephemeris error. In addition, these techniques used in deformation monitoring are criticized as being “point” sensing since they can only collect displacements of a few predesigned points. In practical monitoring, these “point” sensing techniques may not be able to observe damage that has occurred in other positions if there are not enough sensors. However, installing a large number of “point” sensors to obtain distributed deformation may result in a significant increase to cost for long-term monitoring. Replacing these “point” sensors with some kind of distributed sensor can provide a new way to have comprehensive monitoring at an acceptable cost.

In recent years, some methods including slope variation [8] and strain distribution [9] are used before or after loads are applied to calculate bridge deformation indirectly. Considering that the slope is the first derivative of bending deflection in a Euler–Bernoulli beam, an *n* degree polynomial is used to approximate to the bridge deflection that can be differentiated once to an *n* − 1 degree polynomial. Then, the *n* monitored slopes and their position coordinates can be substituted to the *n* − 1 degree polynomial to form the *n* − 1 degree polynomial equations. The solution of the equations gives the bridge deflection. This method is only applicable to small and single-span bridges. In the case of a long-span continuous beam bridge, it is still required to deploy a large number of expensive and high-precise inclinometers. The double integration method (DIM) can also achieve the bending deflection curve of a Euler–Bernoulli beam by double integrating strain distribution and the cost of the distributed strain sensors is lower than that of the high-precision inclinometers. The results from model tests of a simple-supported beam show that the maximum difference between the monitoring displacement and the true value is only about 3% [10,11,12]. However, according to data from a practical deflection monitoring on a multi-span beam bridge, the monitoring error in the second span can rise to over 15% and is significantly higher than the difference of about 3% in the first span [13,14]. This error increased because measurement errors accumulate in the double integrating process. To solve this problem, an improved conjugated beam method (ICBM) has been proposed to deduce the influence of error accumulation [15].

The mentioned methods for deflection monitoring are suitable for a solid rectangular beam suffering a bending moment, but their use may not be suitable for a box girder—a typical hollow beam widely employed in long-span bridges. The box girder has two additional deformations caused by the shear lag effect and shearing action. Similar results from different researchers [16,17,18] have illustrated that the first additional deformation (AD1) caused by the shear lag effect at the mid-span of a simply supported box girder can approach 10% of the bending deflection when the height–span ratio exceeds 0.1. The second additional deformation (AD2) caused by the shear action can also reach 10% of the bending deflection when the shear span–depth ratio is lower than 1/20 [19]. The existing methods based on the distributed strain measurements of a Euler–Bernoulli beam including DIM and ICBM should be revised for a box girder to obtain AD1 or AD2.

There is a challenge to obtaining enough strain data while trying to minimize the number of strain sensors to cover the entire box girder. For this problem, the fiber Bragg grating (FBG) sensor may be an acceptable solution to obtain the strain distribution due to their ability to be linked on a common optical fiber that reduces the difficulties in sensor installation and improved maintenance cost. In recent years, many in-service bridges have installed structural health monitoring systems based on FBG sensors to observe the long-term strain–stress variation and vibration [20,21,22], and monitor bridge scour [23]. Similar FBG-based applications are reported in strain monitoring [24,25,26] for leakages in pipelines, scour monitoring [27], and concrete deterioration and reinforcement corrosion [28,29,30]. A long-gage fiber Bragg grating (LFBG) strain sensor is especially suitable for practical strain monitoring because it allows for the entire structure to be covered by a limited number of sensors. The most notable advantage of the LFBG sensor is that it can measure the average strain of a long distance, from 0.1 m to 10 m. A packaged design of the LFBG sensor [31] and sensitivity-improved LFBG sensor [32] have been proposed for practical monitoring of the slight strain variations. The LFBG sensor has also been verified to be in measuring dynamic strain [33], dynamic displacement [34], and detecting the neutral axis position and damage [35]. Therefore, the LFBG sensor is seen as a useful tool for high-precision strain measurement with a relatively low cost.

This paper is organized as follows. Section 2 introduces the ICBM as proposed in our previous research and the LFBG strain sensor that is used in the experiments. Section 3 revises the ICBM to allow for monitoring AD1 and AD2 based on long-gage strain measurements. Section 4 gives an applicable solution for LFBG sensor placement for the practical monitoring of a single-cell box girder. Finally, experiments using numerical models and a reduced-scale concrete box girder monitored by LFBG sensors are shown in Section 5 to evaluate the theoretical and actual precision of the revised ICBM.

## 2. ICBM and LFBG Sensors

### 2.1. Improved Conjugated Beam Method

**Figure 1:**
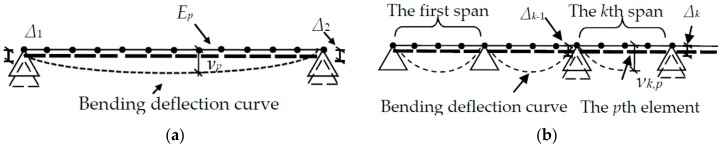
Schematic diagram of: (**a**) simply-supported solid beam; (**b**) continuous multi-span beam.

As the long-gage strains are of interest, ICBM [15] is used to provide a linear and explicit relationship between the bending deflection and strain distribution. This explicit relationship in ICBM is advantageous compared to the DIM that only gives an implicit relationship and double-integrated function. Therefore, in ICBM, it is easy to predict error accumulation from each monitoring parameter. A simply-supported solid beam is shown in Figure 1a. It has length *L* and uniform flexural rigidity *EI*. The beam is uniformly divided into *m* elements artificially, denoted as *E*_1_~*E_m_*. The height of the beam is *h*. The average strains at the top surface and the bottom surface of *E_i_* are ${\bar{\varepsilon }}_{i}^{B}$ and ${\bar{\omega }}_{i}^{B}$ (1 ≤ *i* ≤ *m*), respectively. Superscript B implies that the variable is used under the pure bending.

Without any support settlement, the vertical displacement $\nu_{p}^{B}$ at the boundary point between *E_p_* and *E_p_*_+1_ is:

(1) $$v_{p}^{B}=-\frac{L^{2}}{m^{2}}\left[\frac{p}{m}\sum_{i=1}^{m}{\bar{\kappa }}_{i}^{B}\left(m-i+\frac{1}{2}\right)-\sum_{i=1}^{p}{\bar{\kappa }}_{i}^{B}\left(p-i+\frac{1}{2}\right)\right],$$

where ${\bar{\kappa }}_{i}^{B}$is the average curvature of *E_i_* that can be calculated by Equation (2) below. Tensile strain and upward deflection are defined to be positive in this paper:

(2) $${\bar{\kappa }}_{i}^{B}=\frac{{\bar{\omega }}_{i}^{B}-{\bar{\varepsilon }}_{i}^{B}}{h}.$$

Considering the combined action of arbitrary loads and support settlements, the vertical displacement $\nu_{p}^{B}$ at the boundary point between *E_p_* and *E_p_*_+1_ can be revised as follows:

(3) $$v_{p}^{B}=-\frac{L^{2}}{m^{2}}\left[\frac{p}{m}\sum_{i=1}^{m}{\bar{\kappa }}_{i}^{B}\left(m-i+\frac{1}{2}\right)-\sum_{i=1}^{p}{\bar{\kappa }}_{i}^{B}\left(p-i+\frac{1}{2}\right)\right]+\frac{m-p}{m}\Delta_{0}+\frac{p}{m}\Delta_{1},$$

where Δ_0_ and Δ_1_ are two support settlements, respectively, that can be measured by displacement meters.

ICBM can be adapted to monitor bending deflection for a multi-span bridge as shown in Figure 1b. For a multi-span bridge, Δ*_k_*_−1_ and Δ*_k_* represent the settlements that have occurred at two supports of the *k*th span of the bridge. The *k*th span is equally divided into *m* elements labelled as *E_k,_*_1_~*E_k,m_*. The length and the height of the *k*th span are *L_k_* and *h_k_*, respectively. The average strains at the top surface and the bottom surface of the *E_k,i_* are ${\bar{\varepsilon }}_{k,i}^{B}$ and ${\bar{\omega }}_{k,i}^{B}$ (1≤ *i* ≤ *m*), respectively. Thus, the vertical displacement $\nu_{k,p}^{B}$ at the boundary point between *E_k,p_* and *E_k_*_,*p*+1_ in the *k*th span is:

(4) $$v_{k,p}^{B}=-\frac{L_{k}^{2}}{m^{2}}\left[\frac{p}{m}\sum_{i=1}^{m}{\bar{\kappa }}_{k,i}^{B}\left(n-i+\frac{1}{2}\right)-\sum_{i=1}^{p}{\bar{\kappa }}_{k,i}^{B}\left(p-i+\frac{1}{2}\right)\right]+\frac{m-p}{m}\Delta_{k-1}+\frac{p}{m}\Delta_{k},$$

where ${\bar{\kappa }}_{k,i}^{B}$ is the average curvature of *E_k,i_* that can be calculated by Equation (5):

(5) $${\bar{\kappa }}_{k,i}^{B}=\frac{{\bar{\omega }}_{k,i}^{B}-{\bar{\varepsilon }}_{k,i}^{B}}{h_{k}}.$$

From Equation (1) to Equation (5), two remarkable features of ICBM are summarized as follows. The first feature is that the formula of ICBM is linear and explicit. All parameters are free from actual load patterns or flexural rigidity of the monitored beam. The second feature is that the precision of bending deflection monitoring in one span is only influenced by the measurement errors of strain distribution in the same span. Consequently, the measurement error accumulation of one span does not affect the monitoring results of bending deflection in other spans.

The ICBM is effective for a basic assumption of “solid beam”. With this assumption, the beam has enough rigidity to retain its shape regardless of the loading mode (LM). However, a minor change to the cross-section shape of box girder under the action of a changing LM requires a revision to the ICBM.

### 2.2. LFBG Strain Sensor

**Figure 2:**
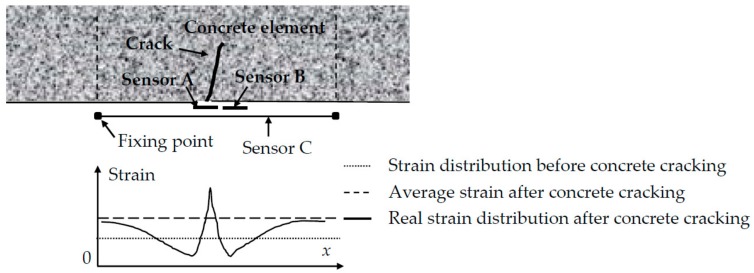
Different strain measurements from three sensors before and after concrete cracking.

In concrete structures, precise and long-term strain monitoring is quite difficult due to concrete cracking. Figure 2 illustrates the traditional strain measuring by short-gage sensors (Sensor A and Sensor B) that are bonded to the surface of the structure using resin. Before concrete cracking, both sensors measure the true concrete strain. After a crack occurs, Sensor A may be overstretched to the point where it may even break because its gauge covers the crack. The strain measured in Sensor B is almost released fully at the same time. The measurements from such short-gage sensors cannot represent the true strain increasing in this case. Long-gage sensors address the point fixation concern as only the two ends of the sensor are bonded to the specimen to measure the uniform strain distribution in its gauge. Another notable advantage of a long-gage sensor is the fact that the sensor can avoid sudden rupture induced by the concrete cracking because the sensing part of the sensor is not directly fixed to the concrete surface. Therefore, it is found that a long-gage sensor has advantages over the traditional short-gage sensor in the strain monitoring for concrete structures.

**Figure 3:**
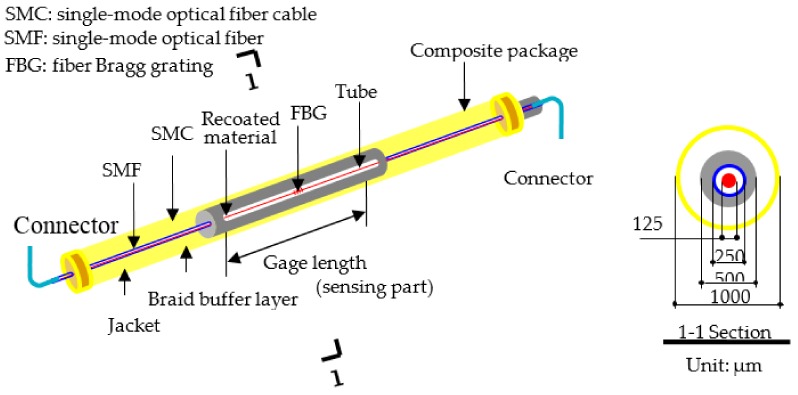
Structural design of packaged long-gage fiber Bragg grating (LFBG) sensor [31].

The monitoring and maintenance costs can be reduced if all sensors can be combined into a sensing network to share the same signal source, connecting wire, and demodulation system. The FBG sensor is characterized by its distributed sensing along a single optical fiber and fulfills the mentioned requirements. Based on an FBG sensor, Li [31] proposed a long-gage fiber Bragg grating (LFBG) sensor that successfully interweaves the precision and distributed sensing characteristic of FBG sensor and the applicability of long-gage sensors in long-term strain monitoring. Considering these benefits, LFBGs shown in Figure 3 are used in this paper to measure long-gage strain in each element of the box girder.

## 3. ICBM Modifications for Single-Cell Box Girders

Results from theoretical derivation and numerical simulation indicate that the total deformation of a box girder can be divided into three parts. These parts are the bending deflection that is the main portion of the total deformation, AD1 caused by the shear lag effect, and AD2 caused by the shear action. As bending deflection is obtained by ICBM as described above, this section derives the relationship between AD1, AD2, and the distributed long-gage strain measurement. The derivations are based on the following assumptions. First, the material used is isotropic and elastic. Second, the stress–strain curve of the material used is linear. Third, the shear lag effect can only affect stress distribution on the cross-section, and it cannot influence the stress distribution in the longitudinal direction. Fourth, the total deformation of the single-cell box girder remains relatively small. Finally, torsion, torsional warping, and distortion are ignored.

### 3.1. AD1 Modification

The shear lag effect represents a phenomenon on the cross-section of box girder where the longitudinal stress on a flange near the web is much larger than that far from the web. This stress distribution is quite different from the uniform stress distribution assumption in the elementary beam theory. This phenomenon also implies that the flange far from the web on the cross-section barely contributes to flexural rigidity. Therefore, there is an additional curvature that occurs on of the section due to the extra decrease in flexural rigidity when calculated according to the elementary beam theory. AD1 is the accumulation of this additional curvature.

Results from numerical simulations [17] show that the practical curvature of the section is equal to the product of *λ* and the curvature as calculated according to the elementary beam theory, where *λ* is the shear lag coefficient defined as the ratio of the normal stresses to those obtained according to the elementary beam theory. This conclusion has two implications. One is that AD1 is a special bending deflection and Equation (1) is applicable to describe the relationship between AD1 and the additional curvature increments in all elements. The second is that the actual measured average strains are also the products of *λ_i_* and strains calculated according to the elementary beam theory and can be shown as ${\bar{\beta }}_{i}$ (${\bar{\beta }}_{i}=\lambda_{i}{\bar{\varepsilon }}_{i}^{B}$) and ${\bar{\alpha }}_{i}$ (${\bar{\alpha }}_{i}=\lambda_{i}{\bar{\omega }}_{i}^{B}$), where *λ_i_* is the shear lag coefficient of *E_i_*. Therefore, the curvature ${\bar{\kappa }}_{i}^{B}$ in Equation (2) has to be replaced by ${\bar{\gamma }}_{i}$ (${\bar{\gamma }}_{i}=\lambda_{i}{\bar{\kappa }}_{i}^{B}$). The superscript SL implies that the variable is used under shear lag action. Thus, the expression of ${\bar{\gamma }}_{i}$ is shown as follows:

(6) $${\bar{\gamma }}_{i}=\lambda_{i}{\bar{\kappa }}_{i}^{B}={\bar{\kappa }}_{i}^{B}+{\bar{\kappa }}_{i}^{\mathrm{SL}}={\bar{\kappa }}_{i}^{B}+(\lambda_{i}-1){\bar{\kappa }}_{i}^{B}=\frac{{\bar{\omega }}_{i}^{B}-{\bar{\varepsilon }}_{i}^{B}}{h}+(\lambda_{i}-1)\frac{{\bar{\omega }}_{i}^{B}-{\bar{\varepsilon }}_{i}^{B}}{h}=\frac{\lambda_{i}{\bar{\omega }}_{i}^{B}-\lambda_{i}{\bar{\varepsilon }}_{i}^{B}}{h}=\frac{{\bar{\alpha }}_{i}-{\bar{\beta }}_{i}}{h},$$

where ${\bar{\kappa }}_{i}^{\mathrm{SL}}$is the additional curvature caused by the shear lag effect.

As a result, the AD1 represented as $\nu_{p}^{\mathrm{SL}}$ at the boundary point between E*_p_* and E*_p_*_+1_ is:

(7) $$\begin{array}{ll} \nu_{p}^{\mathrm{SL}} & =-\frac{L^{2}}{m^{2}}\left[\frac{p}{m}\sum_{i=1}^{m}{\bar{\kappa }}_{i}^{\mathrm{SL}}\left(m-i+\frac{1}{2}\right)-\sum_{i=1}^{p}{\bar{\kappa }}_{i}^{\mathrm{SL}}\left(p-i+\frac{1}{2}\right)\right]\\ & =-\frac{L^{2}}{m^{2}}\left[\frac{p}{m}\sum_{i=1}^{m}(\lambda_{i}-1){\bar{\kappa }}_{i}^{B}\left(m-i+\frac{1}{2}\right)-\sum_{i=1}^{p}(\lambda_{i}-1){\bar{\kappa }}_{i}^{B}\left(p-i+\frac{1}{2}\right)\right]\\ & =-\frac{L^{2}}{m^{2}}\left[\frac{p}{m}\sum_{i=1}^{m}\lambda_{i}{\bar{\kappa }}_{i}^{B}\left(m-i+\frac{1}{2}\right)-\sum_{i=1}^{p}\lambda_{i}{\bar{\kappa }}_{i}^{B}\left(p-i+\frac{1}{2}\right)]+\frac{L^{2}}{m^{2}}[\frac{p}{m}\sum_{i=1}^{m}{\bar{\kappa }}_{i}^{B}\left(m-i+\frac{1}{2}\right)-\sum_{i=1}^{p}{\bar{\kappa }}_{i}^{B}\left(p-i+\frac{1}{2}\right)\right]\\ & =-\frac{L^{2}}{m^{2}}[\frac{p}{m}\sum_{i=1}^{m}{\bar{\gamma }}_{i}\left(m-i+\frac{1}{2}\right)-\sum_{i=1}^{p}{\bar{\gamma }}_{i}\left(p-i+\frac{1}{2}\right)]-\nu_{p}^{B} \end{array}.$$

Consequently, the sum of $\nu_{p}^{B}$ and $\nu_{p}^{SL}$ is given by the following:

(8) $$v_{p}^{B}+v_{p}^{\mathrm{SL}}=-\frac{L^{2}}{m^{2}}\left[\frac{p}{m}\sum_{i=1}^{m}{\bar{\gamma }}_{i}\left(m-i+\frac{1}{2}\right)-{\bar{\gamma }}_{i}\left(p-i+\frac{1}{2}\right)\right].$$

Considering the combined action of arbitrary loads and support settlements, the sum of $\nu_{p}^{B}$ and $\nu_{p}^{SL}$ is revised as follows:

(9) $$v_{p}^{B}+v_{p}^{\mathrm{SL}}=-\frac{L^{2}}{m^{2}}\left[\frac{p}{m}\sum_{i=1}^{m}{\bar{\gamma }}_{i}\left(m-i+\frac{1}{2}\right)-\sum_{i=1}^{p}{\bar{\gamma }}_{i}\left(p-i+\frac{1}{2}\right)\right]+\frac{\left(m-p\right)}{m}\Delta_{0}+\frac{p}{m}\Delta_{1}.$$

### 3.2. AD2 Modification

Before deriving AD2’s expression, it is worth discussing whether shear action causes extra longitudinal strain. It is proposed in material mechanics that the extra longitudinal strain can be ignored in a slender beam subjected to a uniformly distributed load. When the beam is subjected to a concentrated load, the influence in strain from shear action is still approximately zero except for the area close to the supporting points. Therefore, it is reasonable to assume that the longitudinal strain is free from shear action for the purpose of deriving AD2’s expression.

Timoshenko [36] points out that the first derivative of *v*^S^(*x*) with respect to *x* equals the shear strain in the neutral axis of the cross-section. Here, *v*^S^(*x*) represents the AD2 at coordinate *x*. Superscript *S* implies that the variable is used under shear action. Thus, this point can be expressed as follows:

(10) $$\frac{dv^{s}(x)}{dx}=-\frac{\eta V(x)}{AG},$$

where *x* is the longitudinal coordinate, *G* is the shear modulus of the material, *A* is the cross-section area, respectively, *V*(*x*) is the shear force along the section at coordinate *x*, and *η* is the shear correction factor that is equal to the ratio of shear stress *τ*_NA_ on the neutral axis to average shear stress *τ* on the entire section.

Since shear force *V*(*x*) is the first derivative of the moment *M*(*x*), Equation (10) can be transformed into Equation (11) as follows:

(11) $$\frac{dv^{s}(x)}{dx}=-\frac{\eta }{AG}\frac{dM(x)}{dx}.$$

Integrating on both sides of Equation (11) from 0 to *x* and considering both *ν*^S^(0) and *M*(0) equal to 0 in the case of a simply-supported condition, it is obtained as follows:

(12) $$v^{s}(x)=-\frac{\eta }{AG}M(x).$$

Shear correction factor *η* can be expressed by the following:

(13) $$\eta =\frac{\tau_{\mathrm{NA}}}{\bar{\tau }}=\frac{VS_{y}}{2t_{\mathrm{NA}}I\bar{\tau }}=\frac{AS_{y}}{2t_{\mathrm{NA}}I},$$

where *S_y_* is the first moment with respect to the neutral axis of the area on one side of the neutral axis, *V* is the shear force on neutral axis, *I* represents the moment of inertia of the entire cross-sectional area, and *t*_NA_ is the width of web as measured at the same height as the neutral axis.

Substituting Equation (13) into Equation (12) results in the simplified equation:

(14) $$v^{s}(x)=-\frac{S_{y}}{2Gt_{\mathrm{NA}}I}\cdot M(x)=-\frac{(1+\mu )S_{y}}{t_{\mathrm{NA}}}\cdot \frac{M(x)}{EI}=-\frac{(1+\mu )S_{y}}{t_{\mathrm{NA}}}\cdot \kappa^{B}(x)=-\frac{(1+\mu )S_{y}}{\lambda t_{\mathrm{NA}}}\cdot \gamma (x),$$

where *E* and *μ* represent the elastic modulus and Poisson’s ratio of the material, respectively.

Therefore, the AD2 named as $\nu_{p}^{S}$ at the boundary point between *E_p_* and *E_p_*_+1_ is determined from:

(15) $$v_{p}^{S}=-\frac{(1+\mu )S_{y}}{(\lambda_{p}+\lambda_{p+1})t_{\mathrm{NA}}}\cdot ({\bar{\gamma }}_{p}+{\bar{\gamma }}_{p+1}).$$

It is noted that the shear lag coefficient *λ* of each element is predetermined before calculating Equation (15). However, the value of *λ* fluctuates according to different LMs and different positions. In addition, it is difficult to identify the accurate LM in the real structure using practical monitoring. To address these issues, keeping a constant value for *λ* for the entire calculating process is a simple solution. From the literature [16,37], Table 1 gives recommended values of *λ* for a single-cell beam under a simply supported condition.

With a constant *λ*, Equation (15) can be simplified to:

(16) $$v_{p}^{S}=-\frac{(1+\mu )S_{y}}{2\lambda t_{\mathrm{NA}}}\cdot ({\bar{\gamma }}_{p}+{\bar{\gamma }}_{p+1}).$$

Finally, the entire deformation *ν* including the bending deflection, AD1, and AD2 is obtained by the following expression:

(17) $$\upsilon =v_{p}^{B}+v_{p}^{\mathrm{SL}}+v_{p}^{S}=-\frac{L^{2}}{m^{2}}\left[\frac{p}{m}\sum_{i=1}^{m}{\bar{\gamma }}_{i}\left(m-i+\frac{1}{2}\right)-\sum_{i=1}^{p}{\bar{\gamma }}_{i}\left(p-i+\frac{1}{2}\right)\right]-\frac{(1+\mu )S_{y}}{2\lambda t_{\mathrm{NA}}}\cdot ({\bar{\gamma }}_{p}+{\bar{\gamma }}_{p+1})+\frac{\left(m-p\right)}{m}\Delta_{0}+\frac{p}{m}\Delta_{1}.$$

## 4. LFBG Sensor Placement in Box Girders

There are two basic problems in the practical monitoring of a box girder. One is to choose a proper position in the box girder for sensor placement. The other is to determine the minimum number of sensors that can still obtain accurate deformation measurements.

**Figure 4:**
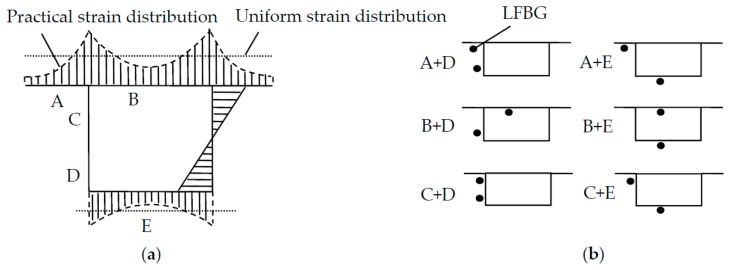
Box girder sensor placements. (**a**) strain distribution on the section; (**b**) six schemes of sensor placement to measure strain distribution.

As shown in Figure 4a, the strain distribution on the flange of a box girder is non-uniform due to the action of shear lag effect. The non-uniformity of strain requires a discussion of the strain sensor placement in the box girder. As illustrated in Figure 4b, there are five possible locations from A to E for the placement of LFBG sensors in addition to two additional concerns that affect strain sensor placement. The first one is that fixing the sensor on the outer surface of a box girder is typically more practical than doing so on the inner surface of a box girder. The other one is that the strain distribution around the practical fixing location needs to follow the plane-section assumption to be far from the influence of shear lag. To address these two concerns, the scheme with sensor placements at C + D is likely the most suitable choice for the LFBG sensor placement in practice.

The minimum number of LFBG sensors is determined according to two considerations. The first consideration is the maximum gage length of the LFBG sensor. The gage length of common LFBG sensor ranges from several centimeters to 1–2 m. The minimum number of LFBG sensors varies based on the gage length of the sensor used and the length of the box girder. The second consideration is the number of elements needed to ensure the measured average strains are close to the true strain in each element. Generally, the number of elements should not be less than 15–18.

## 5. Verification of Revised ICBM: Numerical Simulation

There are two types of errors that can decrease the accuracy of monitored deformation. One is algorithm errors caused by inaccuracy in the simulation for real structures, and the other is measurement errors in the practical strain monitoring. In this verification, a numerical model simulating a real single-cell box girder under different loading modes (LMs) is used to evaluate the influence of algorithm error on the revised ICBM. The evaluation of measurement error accumulation in experimental testing is carried out in Section 6.

### 5.1. Test Design

**Figure 5:**
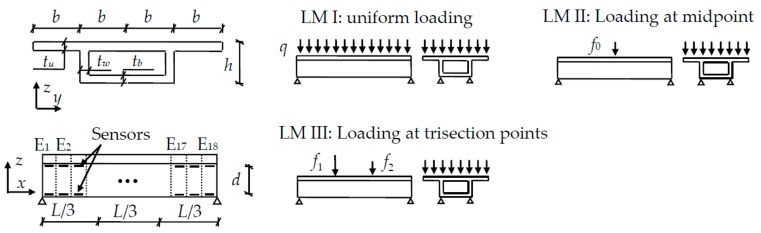
Detailed design for the numerical model of a single-cell box girder for three different loading modes (LMs).

The numerical model is built based on the SOLID45 element in ANSYS software (version, Manufacturer, City, US State abbrev. if applicable, Country). Details of the single-cell concrete box girder with single-supported boundary condition are shown in Figure 5. The dimensions of the cross-section are as follows: *b* = 400 mm, *t_u_* = *t_w_* = *t_b_* = *t*_NA_ = 50 mm, *h* = 300 mm, and *L* = 3600 mm. The compressive strength of concrete is 23.1 N/mm^2^. The elastic module and Poisson’s ratio of concrete are 34.5 GPa and 0.2, respectively. The beam is uniformly divided into 18 elements, denoted as E_1_–E_18_.

There are three different LMs applied to the model. These LMs are uniform loading, loading at the midpoint, and loading at the trisection points. According to Table 1, *λ* is 1.1 since *L*/*b* = 3600/400 = 9. The surface load *q* is 20 kN/m^2^. Linear loads are *f*_0_, *f*_1_, and *f*_2_ that had values of 41.25 kN/m, 41.25 kN/m, and 20.625 kN/m, respectively.

A series of long-gage strain sensors are simulated to be fixed at the two horizontal edges of each element in the web. The distance *d* between the upper part and lower part of sensors is 250 mm.

### 5.2. Results and Discussion

**Figure 6:**
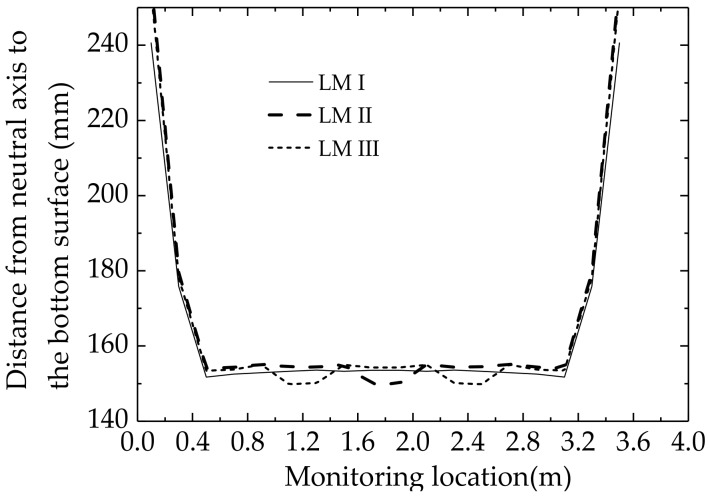
The neutral axis depth of each element for different LMs.

Long-gage strain measurements of 18 elements for the different LMs are given in Table 2. In each element, compressive strain occurs in the upper part, and tensile strain occurs in the lower part. Figure 6 shows the distances between the neutral axis and the bottom surface of each element for three different LMs and where the distance is determined from:

(18) $$\mathrm{Distance}\;\mathrm{in}\;\mathrm{each}\;\mathrm{element}=\frac{{\bar{\alpha }}_{i}/{\bar{\beta }}_{i}}{1+{\bar{\alpha }}_{i}/{\bar{\beta }}_{i}}\cdot d.$$

The neutral axis depths of E3–E16 are almost entirely in the range of 152–153 mm except for some elements near concentrated loads such as E9 and E10 in LM II, and E6, E7, E12, and E13 in LM III. Due to boundary restraints, the tensile strains of E1 and E18 are larger than those of other elements. This causes the neutral axis height for E1, E2, E17, and E18 to be significantly larger than those of other elements. Therefore, the small variation in the height of the neutral axis for the other elements shows the applicability of the plane-section assumption in the web.

**Figure 7:**
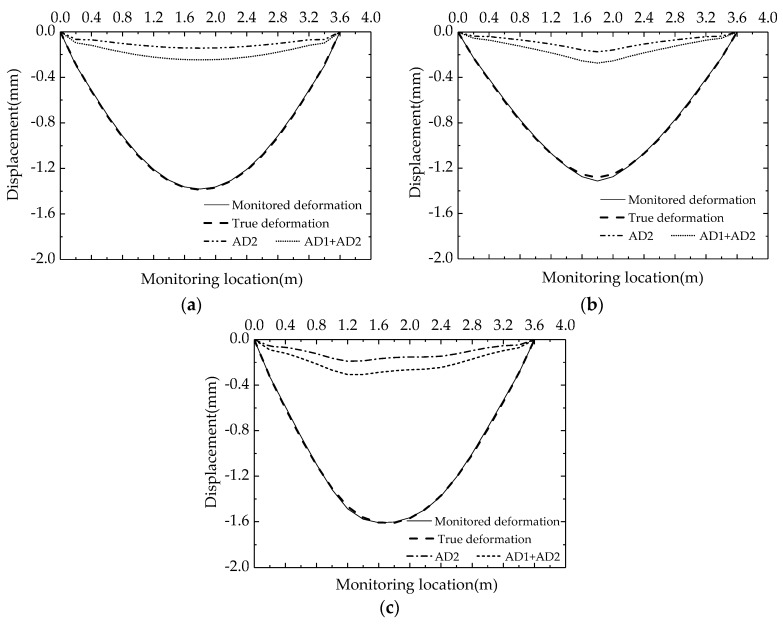
Comparison between monitored deformations and true deformations for different LMs. (**a**) LM I; (**b**) LM II; (**c**) LM III.

A comparison between the monitored deformations and true deformations under the different LMs are shown in Figure 7a–c. The dashed lines represent the true deformations, and the solid lines are the monitored deformations calculated by substituting strain data in Table 2 into Equation (17) (*λ* = 1.1). The dot-dashed lines and the dotted lines in Figure 7 are AD2 and the sum of AD1 and AD2, respectively. It is found that AD1 and AD2 may each separately account for about 10% of the total deformation. Thus, the deformation calculated by the original method shown in Equation (1) is only able to represent about 80% of the total deformation. The difference between the two monitored deformations cannot be ignored, and the proportions of AD1 and AD2 also show that they should be considered separately as in the revised model. Most of the deviations between monitored deformations and true deformations are about 0.3–1.5% except those of E9 and E10 in LM II that had a value of 2.4%. In fact, the accurate value of *λ* under the condition of uniform loading is about 1.069–1.079; this value is close to the assumed constant value of 1.10. The accurate value of *λ* in E9 and E10 under the condition of concentrated loading is 1.266 and is significantly larger than 1.10. This deviation may be the main reason leading to the error of 2.5% in the calculated deformation of E9 and E10 in LM II.

Table 3 lists the monitoring errors between monitoring displacements and true displacements at the 1/3 span, mid span, and 2/3 span. The monitoring errors may be slightly reduced if *λ* is substituted with its accurate value. The maximum error decreased from 2.4% to 0.6% at the mid span in LM II. As the LM in an actual bridge is usually difficult to measure, it is acceptable to have a constant value of *λ* since there is little influence to the precision of deformation monitoring for a single-cell box gilder.

## 6. Verification of Revised ICBM: Experiment

The experiment in this study has two main purposes. One is to show the effectiveness of the revised ICBM to obtain accurate deformation in an actual single-cell concrete box girder. The other is to investigate the possibility of replacing the true *λ* with a constant value in a practical application of the model.

### 6.1. Test Setup and Sensor Placement

**Figure 8:**
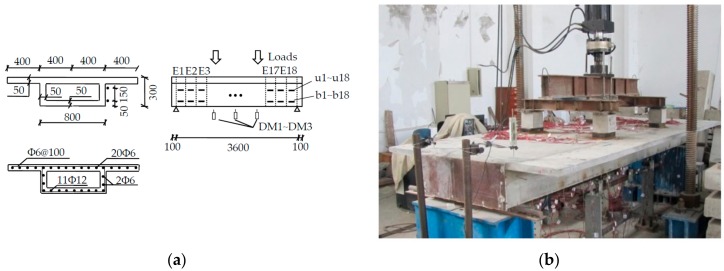
Experiment setup. (**a**) design for the single-cell box girder with sensors placement (unit: mm); (**b**) photograph of the experimental setup.

Details of the beam dimensions and reinforcement configuration of the simply-supported single-cell box girder used in the experiment are shown in Figure 8. The dimensions of the section and the length of the beam are the same as the simulated model shown previously in Figure 5. The compressive strength of concrete used is approximately 39 N/mm^2^. The elastic modulus and Poisson’s ratio of the concrete are 3.03 × 10^4^ N/mm^2^ and 0.19, respectively. In Figure 8, 20 reinforcements that are 6 mm in diameter and 11 passive reinforcements that are 12 mm in diameter are used for longitudinal bars located 20 mm away from the edges of the beam. There are also two passive reinforcements of 6 mm in diameter in each web of the beam. Stirrups are deployed throughout the entire length of the beam of 6 mm diameter and with 100 mm between two adjacent vertical bars. The yield strength of the bars is about 380 N/mm^2^. All mentioned material parameters are determined by standard experiments. Moreover, there are four steel baffles placed at the two trisection points and two ends of the beam to prevent torsional warping and distortion.

The single-cell box girder is divided into 18 zones with a uniform length of 200 mm denoted as Element 1 to Element 18 (E1–E18). There are 36 LFBG sensors with a uniform length of 180 mm installed on the surface of one web of the beam. Half of the LFBG sensors are named as b1–b18 and are fixed at a position 50 mm higher than the bottom of the beam. By contrast, the other half of the LFBG sensors are named as u1–u18 and are fixed at the position 200 mm higher than the bottom of the beam. There is a distance of 150 mm between the two parts of the sensors. Three displacement meters (DM1~DM3) are installed at the two trisection points (point A, point C) and the midpoint (point B) of the beam. Point A, B, and C are the boundary points between E6 and E7, E9 and E10, and E12 and E13, respectively.

The load is divided equivalently into two parts by using a transferred steel board landing at two points 1200 mm from each support. The increasing load is continuously applied by five successive loading steps (LSs) from 0 to 10 KN, 15 KN, 20 KN, 25 KN and 30 KN. The maximum measured strain is ensured to be lower than the value of 100 με that is considered to be the ultimate tensile strain of the concrete. All strain measurements are revised for temperature compensation.

### 6.2. Results and Discussion

**Figure 9:**
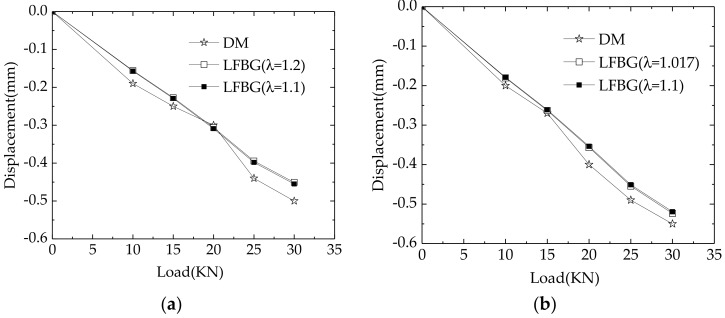

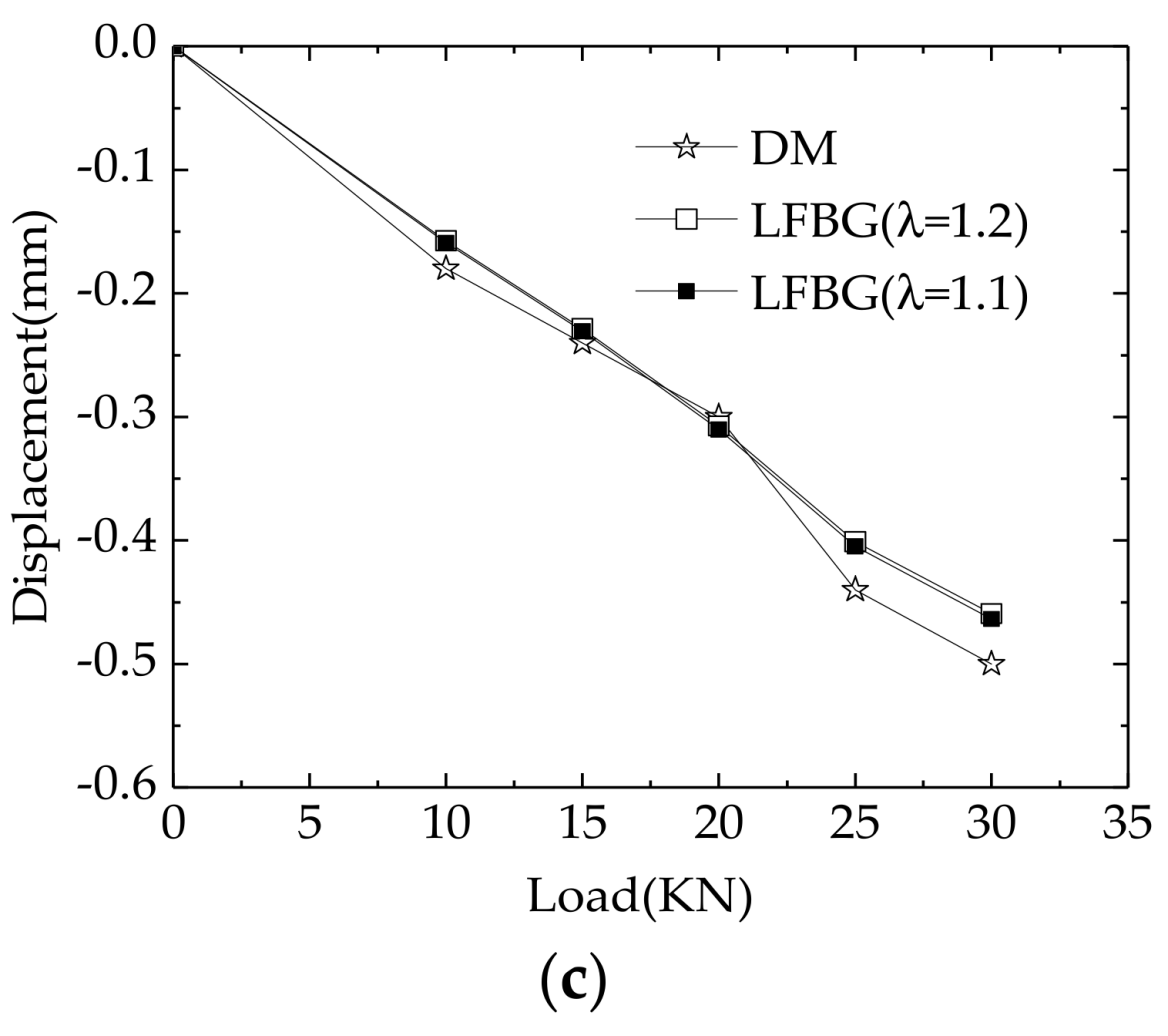
Comparison between monitored displacements and true displacements in different points: (**a**) Point A; (**b**) Point B; (**c**) Point C.

Table 4 gives the long-gage strain measurements from LFBG sensors placed on the surface of each element in different LSs. In each element, the measured strains from b1–b18 are positive, whereas the measured strains from u1–u18 are negative. This illustrates that the single-cell box girder mainly suffers an increasing bending moment with increasing loads. The bending moment brings tension to the lower part of the beam and compression to the upper part of the beam. Figure 9a–c gives the comparisons between the monitored displacements of point A, B, and C for different LSs as calculated from the strain measurements given in Table 4. Upward deformation is defined to be positive. At points A and C, the accurate value of *λ* is 1.2, while at point B it is 1.017. Table 5 shows the comparison of monitoring error percentages between the monitored displacements and the true displacements for each LS. The monitored displacements agree well with the true displacements. Most of the errors ranged from 6–8%, and the maximum monitoring error is only 11.0%. This agreement shows the applicability of the revised ICBM for deformation monitoring of a single-cell box girder based on LFBG sensors. In addition, the fact that the influence from different values of *λ* in deformation monitoring is almost lower than 1% shows that *λ* can be approximated by a constant value according to Table 1 to reduce the difficulty of determining this parameter while ensuring the good accuracy of monitored deformations. A comparison between monitoring errors from Table 3 and Table 5 shows that the precision of monitored deformations is dependent on the precision of the sensors rather than algorithm error. Thus, the algorithm error can be ignored in practical monitoring. In addition, this illustrates the importance of long-term monitoring precision and durability of long-gage sensors in practical deformation monitoring.

## 7. Conclusions

A revised ICBM is proposed in this paper to accurately obtain the entire deformation of a single-cell box girder with a simply-supported boundary condition. A suitable LFBG sensor placement is also proposed for practical monitoring. Verifications using numerical simulations and experiments on a reduced-scale box girder monitored by a series of LFBG sensors are carried out to show the precision of the revised ICBM. From the results and discussions, the following conclusions can be drawn:

(1) For a single-cell box girder, the revised ICBM is verified to be applicable to monitor the entire deformation. The revised ICBM presents a linear and explicit function between the deformation and the long-gage strain distribution. The deformation as considered in the revised ICBM contains the bending deflection, AD1 caused by shear lag, and AD2 caused by the shear action.
(2) The LFBG sensor, a typical long-gage strain sensor, is used to accurately measure the strain distribution on the structural surface and provided a good balance between measurement accuracy and cost.
(3) In the calculations, the shear lag coefficient *λ* can be approximated to be a constant value while still giving good precision in practice of the monitored deformation. Thus, the difficulty of investigating loading mode can be avoided.
(4) Results from numerical simulations show that most algorithm errors are about 0.3–1.5%, and the maximum error is about 2.4%. Results from testing a single-cell box girder monitored by a series of LFBG sensors show that most of the practical errors ranged from 6–8%, and the maximum error is about 11%. Thus, for practical monitoring, the errors in monitored deformation are mainly induced by errors in the strain measurements rather than algorithm error from the revised ICBM.

## Figures and Tables

**Table 1:** Recommended value of *λ* for a single-cell beam under a simply-supported condition [37].

*L*/*b*^1^	6	8	≥10
*λ*	1.22	1.15	1.10

^1^*L* and *b* are the entire length of the beam and the width of flange, respectively.

**Table 2:** Long-gage strains at the top and bottom of each element on the finite element model of the box girder. (Unit: με).

LM		E1	E2	E3	E4	E5	E6	E7	E8	E9	E10	E11	E12	E13	E14	E15	E16	E17	E18
I	${\bar{\beta }}_{i}$	−5	−35	−57	−69	−80	−89	−96	−101	−103	−103	−101	−96	−89	−80	−69	−57	−35	−5
	${\bar{\alpha }}_{i}$	128	83	88	108	126	141	153	160	164	164	160	153	141	126	108	88	83	130
II	${\bar{\beta }}_{i}$	2	−18	−33	−44	−55	−68	−83	−101	−130	−130	−101	−83	−68	−55	−44	−33	−18	2
	${\bar{\alpha }}_{i}$	71	46	53	71	90	110	134	165	195	195	165	134	110	90	71	53	46	73
III	${\bar{\beta }}_{i}$	2	−31	−56	−77	−101	−139	−147	−125	−116	−110	−108	−113	−103	−78	−60	−44	−24	2
	${\bar{\alpha }}_{i}$	119	77	89	123	165	208	221	204	187	179	177	175	159	128	97	71	61	97

**Table 3:** Monitoring errors between monitored displacements and true displacements in three positions. (Unit: %).

LM	Value of *λ*	Positions		
		1/3 Span	Mid Span	2/3 Span
I	*λ* = 1.1	−0.6	−0.4	−0.6
	*λ* = accurate value	−0.5	−0.1	−0.5
II	*λ* = 1.1	−0.3	2.4	−0.3
	*λ* = accurate value	0.5	0.6	0.5
III	*λ* = 1.1	1.5	−0.3	0.3
	*λ* = accurate value	0.3	0.4	−0.6

**Table 4:** Average strain measurements at the bottom and the top of each element. (Unit: με).

Loading Step		E1	E2	E3	E4	E5	E6	E7	E8	E9	E10	E11	E12	E13	E14	E15	E16	E17	E18
1	${\bar{\beta }}_{i}$ ^1^	−2	−2	−2	−3	−5	−2	−3	−6	−5	−6	−3	−2	−5	−3	−2	−2	−3	1
	${\bar{\alpha }}_{i}$	1	3	5	7	10	13	14	13	12	11	13	15	13	11	7	6	3	2
2	${\bar{\beta }}_{i}$	−2	−2	−4	−5	−6	−4	−5	−8	−7	−7	−8	−5	−4	−5	−6	−4	−2	0
	${\bar{\alpha }}_{i}$	1	2	7	11	15	18	20	19	18	17	19	21	19	13	12	7	2	0
3	${\bar{\beta }}_{i}$	−3	−3	−5	−7	−9	−6	−7	−11	−10	−10	−11	−7	−9	−8	−4	−3	−2	−2
	${\bar{\alpha }}_{i}$	2	5	10	14	19	24	26	25	24	25	24	26	24	19	15	9	6	1
4	${\bar{\beta }}_{i}$	−4	−4	−6	−8	−7	−8	−9	−15	−9	−12	−12	−14	−9	−10	−8	−6	−4	−2
	${\bar{\alpha }}_{i}$	4	7	11	21	20	33	35	33	27	33	34	35	28	30	22	10	9	5
5	${\bar{\beta }}_{i}$	−4	−5	−7	−9	−7	−9	−10	−17	−10	−15	−16	−9	−9	−13	−9	−7	−5	−2
	${\bar{\alpha }}_{i}$	6	8	15	22	27	37	40	33	34	38	39	45	36	30	22	15	8	5

^1^${\bar{\alpha }}_{i}$ and ${\bar{\beta }}_{i}$ are the practical strain measurements from LFBG b*i* and LFBG u*i* (*i* = 1~18), respectively.

**Table 5:** Errors between monitored displacements and true displacements at different points. (Unit :%).

Loading Step		1	2	3	4	5
Point A	*λ* = 1.1	−10.2	−8.2	−6.4	−9.5	−9.0
	*λ* = 1.2	−11.0	−9.1	−7.2	−10.4	−9.8
Point B	*λ* = 1.1	−10.5	−3.3	−11.6	−7.9	−5.6
	*λ* = 1.017	−9.8	−2.5	−10.9	−7.2	−4.8
Point C	*λ* = 1.1	−9.1	−3.9	−6.0	−8.0	−7.3
	*λ* = 1.2	−10.0	−4.8	−6.9	−8.9	−8.2
